# Canadian family physicians' and paediatricians' knowledge, attitudes and practices regarding A(H1N1) pandemic vaccine

**DOI:** 10.1186/1756-0500-3-102

**Published:** 2010-04-14

**Authors:** Eve Dubé, Vladimir Gilca, Chantal Sauvageau, Nicole Boulianne, François D Boucher, Julie A Bettinger, Shelly McNeil, Ian Gemmill, France Lavoie, Manale Ouakki

**Affiliations:** 1 Centre de recherche du CHUL-CHUQ, Centre Hospitalier de l'Université Laval, Québec, Canada; 2 Institut National de Santé publique du Québec, Québec, Canada; 3 University of British Columbia, Vaccine Evaluation Center, BC Children's Hospital, Vancouver, British Columbia, Canada; 4 Canadian Center for Vaccinology, Halifax, Nova Scotia, Canada; 5 Kingston, Frontenac and Lennox & Addington Public health Unit, Kingston, Ontario, Canada

## Abstract

**Background:**

One of the main determinants of public immunization success is health professionals' support and recommendations. Little is known about the physicians' level of support and intentions regarding A(H1N1) pandemic influenza vaccination. The aim of this survey was to document Canadian family physicians' and paediatricians' knowledge, attitudes and practices (KAP) as well as their intentions regarding A(H1N1) pandemic influenza vaccines right before the beginning of the largest immunization campaign in Canadian history.

**Findings:**

A self-administered, anonymous, mail-based questionnaire was sent to a random sample of family physicians and to all paediatricians practicing in Canada. All 921 questionnaires received by October 29 2009 were included in the analysis. Between 72% and 92% of respondents agreed with the statements regarding vaccine safety, effectiveness and acceptability. More than 75% of respondents intended to recommend the A(H1N1) pandemic influenza vaccine to their patients and to get vaccinated themselves. The most significant factors associated with the intention to recommend A(H1N1) pandemic vaccines were physicians' intention to be vaccinated against influenza themselves and the perceived acceptability of the vaccine by the vaccinators.

**Conclusions:**

Most Canadian family physicians and paediatricians surveyed were supportive of the A(H1N1) pandemic influenza vaccination before its implementation and large media coverage.

## Findings

In June 2009, the World Health Organization declared an influenza A(H1N1) pandemic[1]. Modelling studies have shown that vaccination is the most effective intervention in reducing A(H1N1) pandemic influenza morbidity, hospitalisation, and mortality [2-4]. It is commonly recognized that the results of an immunization campaign are determined by vaccine characteristics, vaccination campaign timelines and vaccine uptake. However, the success of a new vaccination campaign should not be taken for granted and public acceptance of a new vaccine should not be assumed [5-7]. Health professionals are known to be the most trusted source of information about vaccination in Canada [8] and their recommendation is one of the main determinants of vaccine uptake [9-11]. Both family physicians and paediatricians are involved in routine vaccine delivery and are expected to play a major role in emerging pandemic vaccination campaigns. However, very little data exist about their acceptance of a pandemic vaccine and, to our knowledge, no systematically collected data regarding physician knowledge, attitudes and practice (KAP) on A(H1N1) pandemic vaccine have been reported.

The main aim of this paper is to document Canadian family physicians' and paediatricians' KAP regarding A(H1N1) pandemic influenza and its prevention by vaccination prior to vaccine approval for clinical use and large media coverage of the immunization campaign.

## Methods

A self-administered, anonymous, mail-based questionnaire was sent between August 20 and September 30 2009 to a random sample of 1182 family physicians and to all 1852 Canadian paediatricians. The *Canadian Medical Directory *[12] was used to identify paediatricians and to obtain a random sample of family physicians. This database contains more than 58 000 listings of medical contact information and is updated each year. Based on the analytical framework for immunization programs in Canada [13], the first section of the questionnaire addressed the respondents' KAP regarding routinely used and newly available or candidate vaccines in Canada, including the A(H1N1) pandemic vaccine. Respondents were asked to base their answers on their own knowledge and opinion. No information on A(H1N1) pandemic influenza infection or the vaccine was provided. Descriptive statistics were generated for all variables. Multivariate logistic regressions models were used to determine variables independently associated with the intention to recommend the A(H1N1) pandemic vaccine. Dependent variables were dichotomized: the responses "strongly agree" and "agree" versus all others. Missing responses were excluded from the analysis. Variables associated in the univariate analysis with the intention to recommend the vaccine at p ≤ 0.20 were entered into the multivariate regression models using the stepwise selection technique. Variables were reevaluated in the final model to check for confounding and model fit. A probability level of p < 0.05 based on two-sided tests was considered statistically significant. The collinearity was checked and the adequacy of the model was evaluated by Hosmer and Lemeshow's goodness of fit test. The Statistical Analysis Systems (SAS) software (version 9.1) was used for statistical analysis. The study protocol was approved by the Ethics Board of the Laval University Hospital Center (reference number 126.05.02).

The A(H1N1) pandemic vaccine was approved in Canada at the end of October 2009. Due to important educational efforts undertaken afterwards and their potential impact on physicians' KAB, we have decided to perform two separate analyses. Only questionnaires received before October 29 2009 were included in this analysis.

### Participation

This study is ongoing. All 921 questionnaires received by October 29 2009, after the first questionnaire mailing, were included in this report. After exclusion of 208 physicians no longer practicing or with wrong addresses, the overall participation rate was 31% (18% for family physicians and 39% for paediatricians). Of the 207 family physicians who managed to complete the survey by October 29 2009, 64% spent more than 21 hours a week in outpatient consultation, 51% were men and 58% had been practicing medicine for 20 years or longer. Of the 714 paediatricians who managed to complete the survey by October 29 2009, 52% spent more than 21 hours a week in outpatient consultation, 42% were men and 46% had been practicing for 20 years or longer. Eighty seven percent of family physicians and 59% of paediatricians stated that vaccines were administered at their main practice.

### Knowledge, attitudes and practices regarding A(H1N1) pandemic influenza and its prevention by vaccination

One third of family physicians (33%) and almost half of the paediatricians (46%) had had experience with A(H1N1) pandemic influenza disease before responding to this survey. Overall, 82% of family physicians and 88% of paediatricians estimated that "A(H1N1) pandemic influenza is severe enough to take special precautions to prevent it".

Approximately 80% of both family physicians and paediatricians perceived A(H1N1) pandemic infection as a serious disease, that would occur frequently without vaccination (Table 1). Thirty seven percent of family physicians and 45% of paediatricians strongly agreed that A(H1N1) generates a significant economic burden in Canada. From 72% to 79% of respondents agreed with the statements regarding the usefulness and safety of the A(H1N1) pandemic influenza vaccines. Comparatively, 52% of family physicians and 72% of paediatricians agreed or strongly agreed that seasonal influenza vaccines are very useful to protect children's health. More than 70% of family physicians and paediatricians considered that the A(H1N1) pandemic vaccine would be well accepted by the public and more than 80% that it would be well accepted by the vaccinators. The majority of family physicians (88%) and paediatricians (90%) intended to recommend the A(H1N1) pandemic influenza vaccine to their patients: 30% of family physicians and 43% of paediatricians stated that they would strongly recommend it. When recommending new vaccines, 38% of family physicians and 54% of pediatricians stated being highly influenced by recommendations made by professionals associations. Seventy-seven percent (77%) of family physicians and 83% of paediatricians intended to get vaccinated against A(H1N1) pandemic influenza themselves while 15% of family physicians and 13% of paediatricians were undecided.

**Table 1:** Knowledge, attitudes and practices regarding A(H1N1) pandemic influenza and A(H1N1) pandemic influenza vaccine

	Strongly Disagree/Disagree	Somewhat Disagree	Somewhat Agree	Agree/Strongly Agree
Family Physicans (n = 207)*				
*A(H1N1) pandemic influenza ...*				
Is a **serious **disease	4%	10%	32%	49%
Would occur **frequently **in Canada without vaccination	8%	10%	26%	52%
Generate a **significant economic burden **in Canada	4%	5%	17%	69%
*A(H1N1) pandemic influenza vaccine will be...*				
Safe	5%	10%	35%	38%
Effective	4%	9%	46%	26%
Well accepted by the public	6%	17%	35%	38%
Well accepted by vaccinators	3%	8%	26%	58%
I will recommend A(H1N1) pandemic vaccine to my patients	1%	6%	20%	68%
Paediatricians (n = 714)*				
*A(H1N1) pandemic influenza ...*				
Is a **serious **disease	3%	6%	27%	59%
Would occur **frequently **in Canada without vaccination	6%	6%	24%	59%
Generate a **significant economic burden **in Canada	2%	3%	15%	77%
*A(H1N1) pandemic influenza vaccine will be...*				
Safe	2%	6%	36%	43%
Effective	3%	6%	45%	28%
Well accepted by the public	5%	13%	34%	42%
Well accepted by vaccinators	2%	4%	21%	67%
I will recommend A(H1N1) pandemic vaccine to my patients	2%	3%	15%	75%

*Due to missing responses, row percentages may not add up to 100%.

Finally, 47% of family physicians and 36% of paediatricians considered that their knowledge on the A(H1N1) pandemic influenza vaccine was insufficient, while 28% of family physicians and 30% of paediatricians considered it to be somewhat sufficient.

### Factors associated with intention to recommend A(H1N1) pandemic vaccines

In multivariate analyses, the most significant factors associated with respondents' intention to recommend the A(H1N1) pandemic vaccine were: intention to get vaccinated against A(H1N1) pandemic influenza themselves; belief that A(H1N1) pandemic vaccine would be well accepted by the vaccinators; perceived safety of the A(H1N1) pandemic vaccine; belief that A(H1N1) pandemic influenza generates a significant health and economic burden; perceived usefulness of seasonal influenza vaccination and compliance with professional associations' recommendations when recommending new vaccines (Table 2).

**Table 2:** Variables associated with respondents intention to recommend A(H1N1) pandemic vaccine*

Variables	OR	95% CI	P value
Respondents intention to receive A(H1N1) pandemic vaccine	7.81	4.32-14.14	<0.0001
Belief^§ ^that A(H1N1) pandemic vaccine will be well accepted by vaccinators	6.41	3.89-10.58	<0.0001
Belief^§ ^that A(H1N1) pandemic influenza generate a significant economic burden in Canada	3.28	1.92-5.61	<0.0001
Being influenced by recommendations made by professionals associations when recommending new vaccines	2.32	1.15-4.68	0.02
Belief^§ ^that A(H1N1) pandemic influenza vaccine will be safe	2.30	1.33-3.98	0.003
Belief^§ ^that A(H1N1) pandemic influenza is a serious disease	2.12	1.25-3.57	0.005
Belief^§ ^that seasonal influenza vaccines are very useful to protect children health	2.01	1.21-3.34	0.007

* Multivariate analyses; OR = odds ratio; CI = confidence interval

^§ ^Strongly agree, agree, somewhat agree

### Interpretation

This survey was conducted at a time when relatively little information about the characteristics of the second wave of A(H1N1) pandemic influenza was available and when very limited data about the new A(H1N1) pandemic vaccines' immunogenicity and safety was disclosed. However, more than one out of three respondents had had some experience with cases of A(H1N1) pandemic influenza and the majority of them were willing to recommend the A(H1N1) pandemic vaccine to their patients. More than 75% of the respondents also indicated a willingness to get the vaccine themselves. This proportion is high when compared to the 26% to 61% vaccination coverage rates for seasonal influenza among healthcare workers reported in a recent Canadian study [14].

Few studies have been done to assess healthcare professionals' KAP regarding pandemic influenza and its prevention by immunization [15-17]. Results of an Australian survey conducted in 2007 with a convenience sample of clinical and non-clinical healthcare workers indicated that 86.8% of medical staff respondents considered that a pandemic influenza would be "very serious" if one was to occur. The majority of healthcare workers surveyed in 2007 (77.5%) believed that the vaccine would protect them against pandemic influenza [16]. Results of another survey conducted in May 2009 with 389 hospital healthcare workers (mainly nurses) in Hong Kong indicated that 47.9% of them intended to accept A(H1N1) pandemic vaccination [15].

One internet survey done in April-May 2009 in Canada reported that 32% of the 213 general practitioners surveyed considered A(H1N1) infection to be a serious or extremely serious disease [17]. Four months later, 49% of family physicians and 59% of paediatricians who participated in our survey agreed or strongly agreed that the A(H1N1) pandemic infection was a serious disease (additional 27% to 32% somewhat agreed). Changes in health professionals' perceived seriousness of a pandemic disease might be explained by additional information received and by policy development regarding A(H1N1) influenza prevention over the summer of 2009.

It is of note that physicians' intention to get the vaccine for themselves and the perceived acceptability of the A(H1N1) pandemic vaccine by the vaccinators are the factors the most strongly associated with the intention to recommend the vaccine to their patients. Several studies have shown that the support of an immunization strategy by vaccinators is perceived as a crucial element in health professionals' intention to adhere to it [13,18,19].

Beliefs that A(H1N1) pandemic influenza generates a significant economic burden, beliefs that A(H1N1) pandemic influenza is a serious disease, as well as beliefs in the safety of the A(H1N1) pandemic vaccine, were associated with physicians' intention to recommend the A(H1N1) pandemic vaccine. However, this association was unexpectedly lower when compared to the perceived acceptability of vaccine by vaccinators and personal intention to be vaccinated. Finally, the influence of recommendations made by professional associations on physicians' intention to recommend a new vaccine is consistent with results of previous studies [20-22].

This study has several limitations. First, as in most health professionals' surveys, there is a potential for non participation bias and socially desirable responses. The analysis was limited to the time period before the approval of A(H1N1) pandemic vaccines in Canada and only responses received by October 29 2009 were included. Participation rate for this time period was relatively low. However, respondents' demographics and professional characteristics were similar to recent studies with higher response rates conducted in Canadian family physicians and paediatricians [23-25]. The fact that the questionnaire was anonymous should also diminish socially desirable responses. Second, this study was conducted before the federal government announced the approval of the A(H1N1) pandemic influenza vaccine and attitudes may have changed after that, as demonstrated in a recent UK study [26]. In the latest study in healthcare workers, more respondents indicated willingness to accept stockpiled H5N1 vaccine during a period of high media coverage due to an H5N1 outbreak at a commercial poultry farm than after (63.4%, vs. 51.9%; p = 0.009) [26]. In our survey, between 12% and 15% of respondents did not answer to statements regarding A(H1N1) pandemic influenza vaccine safety and efficacy. This high level of missing responses, as well as the high proportion of relatively neutral answers, is due in our opinion to the respondents' perceived lack of information on these issues. Finally, the question regarding respondents' intention to recommend A(H1N1) pandemic vaccine did not specify the patients' age groups (children versus adults). In our opinion, this could explain, at least partially, some differences observed between family physicians' and paediatricians' intentions to recommend A(H1N1) pandemic vaccine.

In summary, most Canadian family physicians and paediatricians surveyed were supportive of A(H1N1) pandemic influenza vaccination before its implementation and large media coverage. The next phase of the survey was launched one month after the implementation of A(H1N1) pandemic influenza vaccination in Canada. Results of this first phase provide baseline data to serve as comparison for data collected during the vaccination campaign. Analysis of the responses received after October 29 2009 will give us the opportunity to explore the effect of A(H1N1) pandemic vaccine approval as well as additional dissemination of information, including professional associations' recommendations, on physicians' KAP regarding pandemic influenza and its prevention by vaccination.

## Competing interests

This study was financially supported by the Quebec Ministry of Health and Social Services and an unrestricted grant from GlaxoSmithKline. No private company or their employees were involved in study protocol/questionnaire designing, data collection or data analysis and interpretation.

## Authors' contributions

All authors have been involved in the design of the study. ED, VG and CS have drafted the manuscript. MO performed the statistical analysis. All authors have read and approved the final version of the manuscript.

## References

[B1] Chan M World now at the start of 2009 influenza pandemic 2009 http://www.who.int/mediacentre/news/statements/2009/h1n1_pandemic_phase6_20090611/en/index.html 2009-06-11 [cited 2009-10-26]

[B2] Zivkovic Gojovic M Sander B Fisman D Krahn MD Bauch CT Modelling mitigation strategies for pandemic (H1N1) 2009 CMAJ, Early release published at http://www.cmaj.ca October 13, 2009 10.1503/cmaj.091641 PMC2774362 19825923

[B3] Nuno MCG Gumel AB Assessing the role of basic control measures, antivirals and vaccine in curtailing pandemic influenza: scenarios for the US, UK and Netherlands J R Soc Interface 2007 4 505 521 10.1098/rsif.2006.0186 17251132 PMC2373400

[B4] Sypsa V Pavlopoulou I Hatzakis A Use of an inactivated vaccine in mitigating pandemic influenza A(H1N1) spread: a modelling study to assess the impact of vaccination timing and prioritisation strategies Euro Surveill 2009 14 41 19356 19883541

[B5] Maurer J Does receipt of seasonal influenza vaccine predict intention to receive novel H1N1 vaccine: evidence from a nationally representative survey of U.S. adults Vaccine 2009 27 42 5732 4 10.1016/j.vaccine.2009.07.080 19679219 PMC2771376

[B6] Lau JTF Yeung NCY Choi KC Cheng MYM Tsui HY Griffiths S Acceptability of A/H1N1 vaccination during pandemic phase of influenza A/H1N1 in Hong Kong: population based cross sectional survey BMJ 2009 2009 339 b4164 10.1136/bmj.b4164 PMC2768779 19861377

[B7] Seale H McLaws ML Heywood AE Ward KF Lowbridge CP Van D Gralton J MacIntyre CR The community's attitude towards swine flu and pandemic influenza The Medical Journal of Australia 2009 191 5 267 269 19740048 10.5694/j.1326-5377.2009.tb02781.x

[B8] Lagarde F Summary of Public Opinion on Immunization in Canada 2005 Public Health Agency of Canada 19

[B9] Brewer NT Fazekas KI Predictors of HPV vaccine acceptability: A theory-informed, systematic review Preventive Medicine 2007 45 2-3 107 114 10.1016/j.ypmed.2007.05.013 17628649

[B10] Zimet GD Chapter 24: Psychosocial aspects of vaccine acceptability Vaccine 2006 24 Suppl 3 S201 9 10.1016/j.vaccine.2006.06.017 16950008

[B11] Ritvo P A Canadian national survey of attitudes and knowledge regarding preventive vaccines J Immune Based Ther Vaccines 2003 1 1 3 10.1186/1476-8518-1-3 14613575 PMC280696

[B12] 2008 MD Select National - PinPointer CD-ROM Canadian Medical Directory. Scott's Directories http://www.mdselect.com

[B13] Erickson LJ De Wals P Farand L An analytical framework for immunization programs in Canada Vaccine 2005 23 19 2470 6 10.1016/j.vaccine.2004.10.029 15752833

[B14] 2006 Adult National Immunization Coverage Survey, Immunization and Respiratory Infections Division (IRID), Public Health Agency of Canada 2006

[B15] Chor JSY Ngai KLL Goggins WB Wong MCS Wong SYS Lee N Leung TF Rainer TH Griffiths S Chan PKS Willingness of Hong Kong healthcare workers ot accept pre-pandemic influenza vaccination at different WHO alert levels: two questionnaire surveys BMJ 2009 339 b3391 10.1136/bmj.b3391 19706937 PMC2731837

[B16] Seale H Leask J Po K MacIntyre CR "Will they just pack up and leave?" - attitudes and intended behaviour of hospital health care workers during an influenza pandemic BMC Health Services Research 2009 9 30 19216792 10.1186/1472-6963-9-30 PMC2661074

[B17] TNS Canadian Facts. H1N1 flu virus not an extremely serious condition, most doctors say. Toronto, May 2009 http://www.tns-cf.com/news/09.05.29-H1N1-charts.pdf (accessed 2009 October 19)

[B18] Gilca V Setting priorities for new vaccination programs by using public health officers and immunization managers opinions Vaccine 2008 26 33 4204 9 10.1016/j.vaccine.2008.05.061 18582998

[B19] Gilca V Attitudes of nurses toward current and proposed vaccines for public programs: a questionnaire survey Int J Nurs Stud 2009 46 9 1219 35 10.1016/j.ijnurstu.2009.02.013 19349047

[B20] Oram RJ Impact of recommendations to suspend the birth dose of hepatitis B virus vaccine JAMA 2001 285 14 1874 1879 10.1001/jama.285.14.1874 11308401

[B21] Freed GL Reactions of pediatricians to a new centers for disease control recommendation for universal immunization of infants with hepatitis B vaccine Pediatrics 1993 91 4 699 702 8385309

[B22] Raley JC Gynecologists' attitudes regarding human papilloma virus vaccination: a survey of Fellows of the American College of Obstetricians and Gynecologists Infect Dis Obstet Gynecol 2004 12 3-4 127 33 10.1080/10647440400020661 15763912 PMC1784603

[B23] Duval B Vaccination against human papillomavirus: A baseline survey of Canadian clinicians' knowledge, attitudes and beliefs Vaccine 2007 25 45 7841 7 10.1016/j.vaccine.2007.08.041 17923173

[B24] Duval B General practitioners' knowledge and attitudes about cervical cancer screening and HPV vaccination. Poster presentation, 24th International Papillomavirus Conference and Clinical Workshop, Beijing International Convention Center. China 2007

[B25] Gilca V Will Paediatricians vaccinate against an adult disease? Human Papillomavirus vaccination dilemma. Poster presentation, 24th International Papillomavirus Conference and Clinical Workshop, Beijing International Convention Center. China 2007

[B26] Pareek M Clark T Dillon H Kumar R Stephenson I Willingness of healthcare workers to accept voluntary stockpiled H5N1 vaccine in advance of pandemic activity Vaccine 2009 27 2009 1242 1247 10.1016/j.vaccine.2008.12.006 19114078

